# Case Report: Unexpected HBV reactivation upon nucleos(t)ide analogue discontinuation despite 17 years of sustained HBsAg loss post-liver transplantation

**DOI:** 10.3389/fmed.2026.1954979

**Published:** 2026-09-15

**Authors:** Shuchang Shang, Yuexia Liu, Caiyan Zhao

**Affiliations:** Department of Infectious Diseases, Hebei Medical University Third Hospital, Shijiazhuang, Hebei, China

**Keywords:** case report, HBsAg seroclearance, hepatitis b virus reactivation, liver transplantation, nucleos(t)ide analogue withdrawal

## Abstract

Long-term administration of nucleos(t)ide analogues (NAs) has substantially mitigated the risk of post-transplant hepatitis B virus (HBV) recurrence. However, the safety and feasibility of NA withdrawal in liver transplant (LT) recipients, even following prolonged virological suppression and sustained hepatitis B surface antigen (HBsAg) loss, remain poorly defined. We present the case of an 84-year-old male with a >40-year history of chronic HBV infection, who progressed to HBV-related cirrhosis and hepatocellular carcinoma, culminating in a deceased-donor liver transplantation (DDLT) in 2004. Post-transplant HBV prophylaxis consisted of HBIG combined with nucleos(t)ide analogue therapy, followed by long-term entecavir maintenance alongside tacrolimus-based immunosuppression. The patient achieved sustained HBsAg seroclearance and maintained undetectable HBV DNA levels for 17 years. Consequently, ETV was discontinued in June 2021. In October 2022, an asymptomatic elevation of hepatic transaminases was noted, though HBV-specific virological assessments were omitted. By March 2023, further evaluation of persistent transaminase elevation confirmed robust HBV reactivation: HBV DNA surged to 5.10 × 10⁸ IU/mL, accompanied by HBeAg positivity and a quantitative HBsAg level of 98,008 IU/mL (Abbott Architect assay). Salvage therapy comprising ETV and tenofovir alafenamide (TAF) was promptly initiated, yielding rapid virological suppression and biochemical normalization. HBV DNA fell below the lower limit of detection (<20 IU/mL) within 9 months of retreatment (December 2023) and has remained fully suppressed through April 2026. This case emphasizes that fulminant HBV reactivation may transpire following NA cessation in LT recipients, irrespective of protracted virological suppression and stable HBsAg seroclearance. This trajectory reinforces the paradigm that HBsAg loss in the context of chronic immunosuppression reflects functional viral control rather than complete viral eradication. In concordance with major hepatology guidelines, indefinite NA prophylaxis should remain the cornerstone of management for LT recipients with HBV-related disease, precluding routine drug withdrawal.

## 1 Introduction

Chronic hepatitis B virus (HBV) infection remains a leading global etiology of cirrhosis, hepatocellular carcinoma (HCC), and liver-related mortality (5). In patients with HBV-related end-stage liver disease or HCC undergoing liver transplantation (LT), effective antiviral prophylaxis has substantially improved graft and patient survival by mitigating post-transplant HBV recurrence (1, 6). Long-term nucleos(t)ide analogue (NA) therapy, particularly with high-genetic-barrier agents like entecavir (ETV), achieves durable suppression of HBV replication post-transplantation, with a subset of recipients ultimately achieving sustained hepatitis B surface antigen (HBsAg) seroclearance (1, 7).

Despite profound virological suppression, complete viral eradication is rarely, if ever, achieved (3). Even following apparent functional control, residual HBV reservoirs, including covalently closed circular DNA (cccDNA) and other replication-competent viral templates, may persist within hepatic or extrahepatic compartments (8, 9). Consequently, the safety of NA withdrawal remains highly controversial, particularly in LT recipients subjected to lifelong pharmacological immunosuppression (2, 10). In this condition, blunted immune surveillance may facilitate the reactivation of residual replication-competent HBV, precipitating severe recurrence upon NA cessation (11).

Herein, we report a case of exceptionally late and robust HBV recurrence following the withdrawal of long-term NA prophylaxis in an LT recipient who had maintained undetectable serum HBV DNA and sustained HBsAg loss for 17 years. This case underscores that sustained HBsAg seroclearance does not equate to treatment-free safety in chronically immunosuppressed LT recipients.

## 2 Case presentation

An 84-year-old male presented to our center in March 2023 for the evaluation of persistent hepatic biochemical derangements. His medical history was notable for a > 40-year duration of chronic HBV infection (HBsAg positivity first documented in 1984), progressing to HBV-related cirrhosis in 2001. He underwent sequential antiviral regimens, including interferon, lamivudine, and adefovir rescue therapy following a lamivudine resistance-associated YIDD mutation breakthrough. Immediately before liver transplantation in 2004, laboratory evaluation demonstrated mildly decreased serum albumin, elevated total bilirubin (40.23 μmol/L), with normal alanine aminotransferase (ALT) and aspartate aminotransferase (AST) levels. HBV serological testing showed positive HBsAg, anti-HBe, and total anti-HBc, with negative HBeAg. Serum HBV DNA remained detectable at 3.85 × 10^3^ IU/mL.

In 2004, the patient developed multinodular HCC (Child-Pugh class B) secondary to HBV cirrhosis. He subsequently underwent a deceased-donor orthotopic liver transplantation in June 2004, with complete removal of the native liver. The donor tested negative for HBsAg, anti-HBs, anti-HBc, HBeAg, and anti-HBe, and donor HBV DNA was below the assay detection limit. Explant pathology confirmed two well-differentiated (Edmondson grade II) HCC lesions (1.5 cm and 2.5 cm) arising within a mixed nodular cirrhotic background.

Following transplantation, HBV prophylaxis consisted of hepatitis B immunoglobulin (HBIG) in combination with nucleos(t)ide analogue therapy. According to the available medical records, intravenous HBIG was administered at 2,000 IU/day on the day of transplantation and for the following four postoperative days. During subsequent maintenance therapy, HBIG was administered intramuscularly at 400 IU twice weekly in combination with entecavir (ETV). The patient also received tacrolimus-based immunosuppression. During the early post-transplant period, tacrolimus was administered at 2 mg once daily in combination with methylprednisolone 4 mg once daily and mycophenolate mofetil 500 mg once daily. Methylprednisolone and mycophenolate mofetil were discontinued three months after transplantation, whereas tacrolimus was continued as long-term maintenance immunosuppression.

During long-term post-transplant follow-up, the patient maintained HBsAg seroclearance and undetectable serum HBV DNA for 17 years under antiviral prophylaxis. Anti-HBs was detectable during earlier follow-up but gradually declined and became negative after March 2016. HBIG was discontinued in August 2017 after prolonged virological suppression, whereas ETV therapy was continued. The tacrolimus dose was gradually reduced during follow-up as part of routine post-transplant management and had been maintained at 0.25 mg twice daily since October 2020.

Immediately before ETV withdrawal in June 2021, the patient remained HBsAg negative (0.04 IU/mL), with undetectable HBV DNA (<20 IU/mL), negative HBeAg (0.36 S/CO), negative anti-HBs (1.61 mIU/mL), and persistent total anti-HBc positivity. Following multidisciplinary assessment and shared decision-making, ETV was electively discontinued in June 2021. At that time, tacrolimus remained unchanged at 0.25 mg twice daily, with a trough concentration of 2.31 ng/mL.No adjustment of the immunosuppressive regimen was made around the time of ETV discontinuation. Representative longitudinal HBV serological and virological measurements obtained during post-transplant follow-up before the 2023 recurrence are summarized in Table 1.

**Table 1:** Longitudinal virological and serological profile during post-transplant follow-up before the 2023 HBV recurrence.

Date	HBV DNA (IU/mL)	HBsAg (IU/mL)	Anti-HBs (mIU/mL)	HBeAg (S/CO)	Anti-HBe (S/CO)	Anti-HBc (S/CO)
2004-12-21	<1 × 10^3^	0.05	80.00	0.56	0.21	7.3
2005-12-24	<1 × 10^3^	—	79.00	—	—	—
2006-08-20	<1 × 10^3^	—	81.32	—	—	—
2007-02-21	<1 × 10^3^	—	82.21	—	—	—
2008-12-02	<1 × 10^3^	—	76.96	—	—	—
2009-11-03	—	—	103.47	—	—	—
2010-05-11	<1 × 10^3^	0.03	62.26	0.29	0.42	6.7
2011-02-16	—	—	87.82	—	—	—
2012-06-27	<1 × 10^3^	—	66.49	—	—	—
2013-09-25	<5 × 10^2^	—	63.58	—	—	—
2014-11-13	<1 × 10^3^	0.04	144.10	0.13	0.32	9.5
2015-08-18	<5 × 10^2^	—	159.80	—	—	—
2016-03-31	—	—	125.79	—	—	—
2017-12-13	<20	0.05	2.84	0	0.43	8.3
2018-03-01	<20	0.03	3.43	0.49	0.45	7.62
2019-02-21	<20	—	4.03	—	—	—
2020-05-04	<20	0.04	3.22	0.32	0.36	6.91
2021-06-26	<20	0.04	1.61	0.36	0.33	7.15
2021-11-01	<20	—	—	—	—	—

Representative annual assessments were selected when available during periods of stable follow-up, with additional measurements included around clinically relevant time points.

HBsAg, hepatitis B surface antigen; anti-HBs, antibody to hepatitis B surface antigen; HBeAg, hepatitis B e antigen; anti-HBe, antibody to hepatitis B e antigen; anti-HBc, antibody to hepatitis B core antigen; HBV DNA, hepatitis B virus DNA; —, data not available.

In October 2022, asymptomatic elevations of alanine aminotransferase (ALT, 93 U/L) and aspartate aminotransferase (AST, 63 U/L) were initially noted; however, HBV-specific virological markers were inadvertently omitted from this assessment. The virological relapse remained unconfirmed until March 2023, when the patient was referred for persistent transaminemia.

Laboratory evaluation in March 2023 revealed: ALT 53 U/L, AST 171 U/L, total bilirubin 23.49 μmol/L, albumin 46.34 g/L, alkaline phosphatase 127 IU/L, gamma-glutamyl transferase 50 IU/L, and an estimated glomerular filtration rate of 43.25 mL/min/1.73 m^2^. Virological testing confirmed a robust HBV recurrence: HBV DNA surged to 5.10 × 10⁸ IU/mL, accompanied by HBeAg positivity and a quantitative HBsAg level of 98,008 IU/mL (Abbott Architect assay). At recurrence, total anti-HBc remained positive and anti-HBs was negative. Tacrolimus was continued at 0.25 mg twice daily, with a trough concentration of 2.32 ng/mL; no corticosteroid pulse therapy or escalation of immunosuppression had occurred before recurrence. Comprehensive clinical and radiological evaluations ruled out HCC recurrence, biliary obstruction, vascular complications, and superimposed infections.

Given the formidable viral load, post-transplant status, historical lamivudine resistance, and compromised renal function, intensive salvage therapy comprising ETV and tenofovir alafenamide (TAF) was immediately instituted. Virological response was rapid: HBV DNA declined to 5.31 × 10⁵ IU/mL by April 2023 and further to 3.85 × 10⁴ IU/mL by May 2023, when HBsAg had decreased to 16,172 IU/mL, accompanied by normalization of ALT. By December 2023, HBV DNA had fallen below the limit of detection (<20 IU/mL), and HBsAg had decreased to 57 IU/mL (Abbott Architect assay). The patient has remained on the ETV + TAF regimen, maintaining durable biochemical normalization and virological suppression with HBsAg level of 32 IU/mL (Abbott Architect assay) through April 2026, with no evidence of HCC recurrence. The clinical timeline is summarized in Figure 1, and the longitudinal virological and biochemical responses after salvage therapy are illustrated in Figure 2.

**Figure 1:**
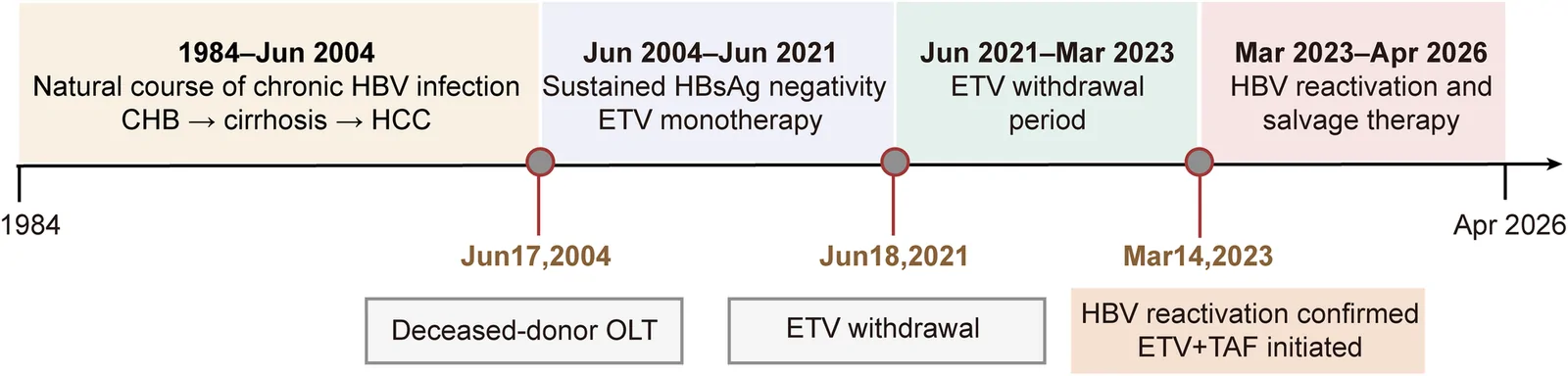
Timeline of the clinical course of a patient with chronic HBV infection before and after liver transplantation.

**Figure 2:**
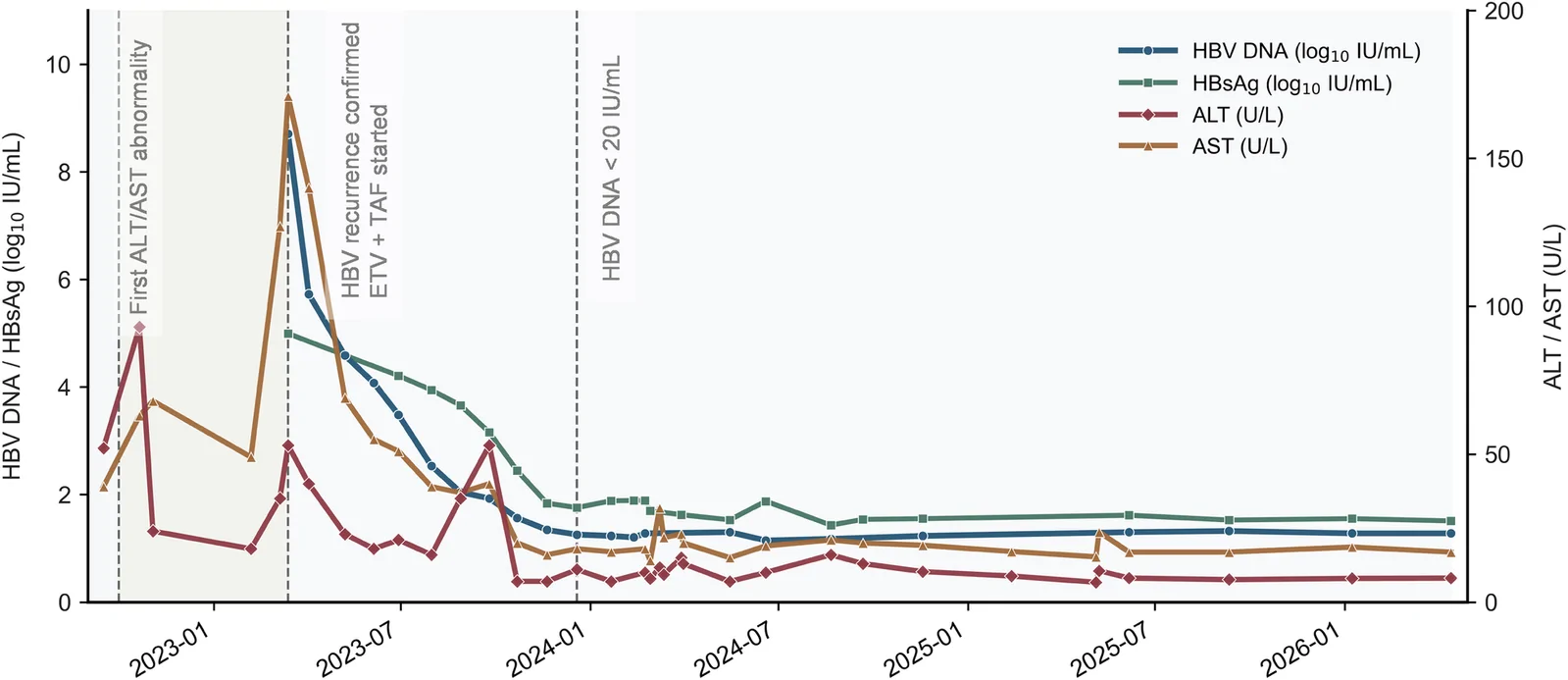
Dynamic changes in virological and biochemical markers after retreatment. Longitudinal changes in serum HBV DNA, HBsAg, ALT, and AST levels following HBV reactivation after nucleos(t)ide analogue withdrawal and subsequent ETV plus TAF therapy. HBV DNA levels are presented as log10 IU/mL.

## 3 Discussion

The clinical trajectory of this patient perfectly illustrates the long-term natural history of chronic HBV infection, highlighting its relentless fibrogenic and oncogenic potential (12). This underscores the unequivocal clinical rationale for indefinite post-transplant antiviral prophylaxis (1, 2, 6, 10).

The advent of high-potency NAs has revolutionized the prevention of post-transplant HBV recurrence. Regimens based on ETV or tenofovir (with or without HBIG) achieve robust viral suppression, and HBsAg seroclearance is frequently observed (1, 7, 13). However, this functional control must not be conflated with a sterilizing virological cure (3, 4, 8, 9). The profound virological rebound observed in our patient—despite 17 years of undetectable HBV DNA and absent HBsAg—strongly suggests that replication-competent HBV reservoirs persist despite protracted serological silencing (8, 9, 13). Notably, sustained HBsAg seroclearance was not accompanied by persistent anti-HBs positivity, as anti-HBs gradually declined and was negative before ETV withdrawal.

Mechanistically, high-genetic-barrier NAs potently inhibit viral reverse transcription but exert minimal efficacy in clearing established intrahepatic cccDNA or integrated HBV DNA sequences (3, 9). Consequently, undetectable serum HBV DNA and HBsAg loss represent milestones of functional viral suppression, not viral eradication (8, 9, 14).

This distinction becomes particularly critical when contemplating prophylaxis minimization in LT recipients, in whom lifelong immunosuppression may impair HBV-specific immune surveillance and facilitate viral recurrence (10, 11). Although HBIG minimization or discontinuation strategies have been explored in selected low-risk LT recipients receiving potent NA therapy, these approaches cannot be extrapolated to justify complete NA cessation, particularly in recipients with HBV-related cirrhosis or HCC requiring long-term immunosuppression (1, 2, 7). In 2004, HBIG combined with nucleos(t)ide analogue therapy represented the standard preventive approach after liver transplantation, particularly during the early post-transplant period (2, 15). In this patient, HBIG was administered in combination with antiviral therapy and was discontinued in August 2017 after prolonged virological suppression, while entecavir therapy was maintained. Importantly, HBV recurrence did not occur after HBIG discontinuation under continued entecavir therapy, but developed after subsequent withdrawal of entecavir. Thus, HBIG discontinuation under continued NA suppression should be clearly distinguished from complete cessation of antiviral prophylaxis (2, 7). Notably, clinical studies exploring NA withdrawal have predominantly focused on carefully selected, immunocompetent, non-cirrhotic HBeAg-negative individuals (16). However, these findings may not be directly applicable to LT recipients, who have a history of HBV-related end-stage liver disease or malignancy, harbor an allograft under chronic immunosuppression, and often require prolonged antiviral therapy to maintain viral suppression (2, 7).

Long-term immunosuppression may have contributed to impaired HBV-specific immune control in this patient (7, 11). Importantly, HBV recurrence was not preceded by intensification of immunosuppressive therapy. The patient remained on a stable tacrolimus-based regimen during the period surrounding entecavir withdrawal and viral recurrence, with tacrolimus trough concentrations of 2.31 ng/mL in June 2021 and 2.32 ng/mL at recurrence in 2023. No corticosteroid pulse therapy or additional immunosuppressive agents were introduced during this period. These findings suggest that persistent immunosuppression provided a permissive environment for HBV persistence, whereas withdrawal of antiviral suppression likely represented the proximate trigger for viral rebound (7, 10, 11).

In this patient, complete removal of the native liver makes persistence within residual native hepatic tissue unlikely, while negative donor HBsAg, anti-HBc, and HBV DNA results argue against donor-derived HBV infection. However, liver transplantation does not necessarily eliminate all HBV reservoirs. This setting differs from mother-to-child transmission prophylaxis, in which HBIG is administered around the time of viral exposure before persistent infection is established (17, 18). In contrast, this patient had longstanding chronic HBV infection before transplantation; therefore, HBIG could reduce circulating virus and protect the graft from infection but would not be expected to eradicate pre-existing intracellular or extrahepatic HBV reservoirs (19). Previous studies have suggested that HBV-related viral materials may persist in extrahepatic compartments even after removal of the infected liver, supporting the possibility of long-term viral reservoirs beyond hepatic tissue (10, 20). Such extrahepatic reservoirs or residual replication-competent HBV populations may contribute to re-establishment of infection in the graft, particularly under long-term immunosuppression.

Several findings support HBV reactivation rather than *de novo* infection. The patient had well-documented chronic HBV infection before transplantation, including HBV-related cirrhosis and hepatocellular carcinoma, HBsAg positivity, and detectable HBV DNA. Total anti-HBc remained positive during post-transplant follow-up, and marked HBV rebound occurred after withdrawal of long-term entecavir therapy. In contrast, the donor was negative for relevant HBV markers, and no recognized post-transplant HBV exposure or other documented epidemiological risk factor for *de novo* HBV acquisition was identified. Taken together, these findings make reactivation of residual HBV infection established before transplantation the most plausible explanation for the 2023 recurrence (10, 20).

Furthermore, this case provides a stark clinical caveat regarding post-withdrawal surveillance. The detection of elevated transaminases in October 2022 preceded the formal virological diagnosis by five months due to omitted specific testing. In LT recipients with a history of HBV, any unexplained hepatic biochemical abnormality—especially following a reduction in prophylactic intensity—must mandate immediate assessment of HBV DNA and serological markers (1, 2, 7).

This report is not without limitations. As a single case observation, it cannot establish the incidence or definitive predictors of recurrence following NA cessation in LT recipients. Additionally, advanced virological biomarkers [e.g., intrahepatic cccDNA quantification, serum HBV RNA, or HBV core-related antigen (HBcrAg)] were not evaluated prior to withdrawal or at the time of recurrence, precluding a precise characterization of the residual viral reservoir (3, 9, 14). Molecular confirmation of viral origin was not possible because pre-transplant HBV genotype or sequence data were unavailable for comparison; such testing was not routinely performed in clinical practice at the time of transplantation in 2004. Therefore, although the available clinical, serological, and temporal evidence supports HBV reactivation as the most plausible explanation, the viral origin could not be definitively confirmed by molecular comparison. Finally, the delay in virological testing upon initial transaminase elevation may have obscured an even earlier virological relapse.

Nevertheless, the exceptionally prolonged duration of prior virological suppression, juxtaposed with the aggressive virological rebound upon NA withdrawal, provides compelling real-world evidence. It unequivocally reinforces the paradigm that HBsAg loss in chronically immunosuppressed LT recipients reflects functional control, rather than a definitive cure, mandating the continuation of lifelong NA prophylaxis (9).

## Data Availability

The raw data supporting the conclusions of this article will be made available by the authors, without undue reservation.
